# Putative novel *cps* loci in a large global collection of pneumococci

**DOI:** 10.1099/mgen.0.000274

**Published:** 2019-06-11

**Authors:** Andries J. van Tonder, Rebecca A. Gladstone, Stephanie W. Lo, Moon H. Nahm, Mignon du Plessis, Jennifer Cornick, Brenda Kwambana-Adams, Shabir A. Madhi, Paulina A. Hawkins, Rachel Benisty, Ron Dagan, Dean Everett, Martin Antonio, Keith P. Klugman, Anne von Gottberg, Robert F. Breiman, Lesley McGee, Stephen D. Bentley, The Global Pneumococcal Sequencing Consortium, Abdullah W. Brooks, Alejandra Corso, Alexander Davydov, Andrew Pollard, Anna Skoczynska, Bernard Beall, Betuel Sigauque, Deborah Lehmann, Diego Faccone, Ekaterina Egorova, Elena Voropaeva, Eric Sampane-Donkor, Ewa Sadowy, Godfrey Bigogo, Helio Mucavele, Houria Belabbès, Idrissa Diawara, Jennifer Moïsi, Jennifer Verani, Jeremy Keenan, KL Ravikumar, Leonid Titov, Margaret Ip, Maria-Cristina de Cunto Brandileone, Md Hasanuzzaman, Metka Paragi, Naima Elmdaghri, Nicole Wolter, Noga Givon-Lavi, Özgen Köseoglu Eser, Pak Leung Ho, Patrick E. Akpaka, Paul Turner, Paula Gagetti, Peggy-Estelle Tientcheu, Philip E. Carter, Pierra Law, Rama Kandasamy, Rebecca Ford, Sadia Shakoor, Samanta Cristine Grassi Almeida, Samir K. Saha, Sanjay Doiphode, Susan A. Nzenze, Shamala Devi Sekaran, Somporn Srifuengfung, Stephen Obaro, Stuart C. Clarke, Tamara Kastrin, Theresa J. Ochoa, Veeraraghavan Balaji, Waleria Hryniewicz, Yulia Urban

**Affiliations:** ^1^​ Parasites and Microbes, The Wellcome Sanger Institute, Wellcome Genome Campus, Hinxton, Cambridge, UK; ^2^​ Division of Pulmonary Medicine, Departments of Medicine and Microbiology, The University of Alabama at Birmingham, Birmingham, AL, USA; ^3^​ Centre for Respiratory Diseases and Meningitis, National Institute for Communicable Diseases, Johannesburg, South Africa; ^4^​ School of Pathology, Faculty of Health Sciences, University of the Witwatersrand, Johannesburg, South Africa; ^5^​ Malawi-Liverpool-Wellcome-Trust, Blantyre, Malawi; ^6^​ Vaccines and Immunity Theme, MRC Unit, Banjul, The Gambia; ^7^​ Medical Research Council: Respiratory and Meningeal Pathogens Research Unit, University of the Witwatersrand, Johannesburg, South Africa; ^8^​ Hubert Department of Global Health, Rollins School of Public Health, Emory University, Atlanta, GA, USA; ^9^​ Faculty of Health Sciences, Ben-Gurion University of the Negev, Beersheeba, Beer-Sheva, Israel; ^10^​ Queens Research Institute, University of Edinburgh, Edinburgh EH8 9YL, UK; ^11^​ Centers for Disease Control and Prevention, Atlanta, GA, USA; ^12^​ Emory Global Health Institute, Emory University, Atlanta, GA, USA; ^13^​ https://www.pneumogen.net/gps/

**Keywords:** *Streptococcus pneumoniae*, pneumococcus, serotype, *cps *locus, polysaccharide capsule

## Abstract

The pneumococcus produces a polysaccharide capsule, encoded by the *cps* locus, that provides protection against phagocytosis and determines serotype. Nearly 100 serotypes have been identified with new serotypes still being discovered, especially in previously understudied regions. Here we present an analysis of the *cps* loci of more than 18  000 genomes from the Global Pneumococcal Sequencing (GPS) project with the aim of identifying novel *cps* loci with the potential to produce previously unrecognized capsule structures. Serotypes were assigned using whole genome sequence data and 66 of the approximately 100 known serotypes were included in the final dataset. Closer examination of each serotype’s sequences identified nine putative novel *cps* loci (9X, 11X, 16X, 18X1, 18X2, 18X3, 29X, 33X and 36X) found in ~2.6  % of the genomes. The large number and global distribution of GPS genomes provided an unprecedented opportunity to identify novel *cps* loci and consider their phylogenetic and geographical distribution. Nine putative novel *cps* loci were identified and examples of each will undergo subsequent structural and immunological analysis.

## Data Summary

1. Supplementary files 1 and 2 (available in the online version of this article).

2. Raw sequencing data for all study genomes have been deposited in the European Nucleotide Archive (ENA). Accession numbers are detailed in Table S1.

3. Nucleotide sequences for each putative novel *cps* locus have been deposited in GenBank (Accession numbers MK606429–MK606437).

**Impact Statement**

The antigenic polysaccharide capsule enclosing the cell wall is the primary pneumococcal virulence factor, protects against phagocytosis by the human immune system and is encoded by the *cps* locus. Nearly 100 capsular types or serotypes have been described to date, but new serotypes continue to be discovered especially in previously understudied regions. We used the largest ever pneumococcal genome collection of over 18 000 genomes to screen for putatively novel *cps* loci that could potentially code for novel serotypes. We identified nine putatively novel *cps* loci in a variety of pneumococcal lineages and in a number of different countries across the globe. Pneumococcal isolates with each of the putative novel *cps* loci will be sent for biochemical and serological characterization to establish whether they encode novel polysaccharides. This study provides a major advance in our understanding of the potential undiscovered serotype diversity in a globally important pathogen whose primary vaccine strategy is the targeting of particular serotypes. The work will be of broad interest to microbiologists, epidemiologists and researchers studying the impact of pneumococcal vaccines on the global pneumococcal population.

## Introduction

*Streptococcus pneumoniae* (the pneumococcus) is a Gram-positive human commensal that is also responsible for a significant global disease burden, with pneumococcal-related diseases such as pneumonia and meningitis responsible for an estimated 300 000–500 000 deaths in children <5 years of age each year in the pre-vaccine era [1]. The pneumococcus produces an extracellular polysaccharide capsule that provides protection against phagocytosis and is the primary pneumococcal virulence factor [2–4]. With the exception of synthase-dependent serotypes 3 and 37, all serotypes use the Wzy-dependent system to produce the polysaccharide capsule [5]. The capsule is encoded by the CPS synthesis (*cps*) locus situated between the *dexB* and *aliA* genes (the serotype 37 capsule is produced by the *tts*, located outside the *cps* locus) and approximately 100 capsular types (serotypes) have been identified, with novel serotypes still being discovered [6–8]. A comparative analysis of 90 *cps* loci showed that there is an extensive repertoire of ~2000 coding sequences that can be divided into three functional groups: common modulatory genes (*wzg*, *wzh*, *wzd*, *wze*) at the 5′ end of the *cps* locus that are unlikely to alter the polysaccharide structure, serotype-specific genes (consisting mainly of different glycosyltransferases and acetyltransferases) and sugar synthesis genes required for capsule production typically found at the 3′ end of the *cps* locus [5, 9, 10]. Capsular gene acquisition from other pneumococci as well as other streptococci such as *S. mitis* and loss of gene function may have caused the emergence of novel serotypes [11, 12].

Safe, effective conjugate vaccines that target the capsular polysaccharide have been developed and administered globally since 2000; however, these vaccines are limited in their serotype valency as they are designed to target only the 10–13 serotypes most prevalent in invasive pneumococcal disease (IPD). Whilst pneumococcal conjugate vaccines (PCVs) have had a significant effect on the prevalence of IPD caused by vaccine-types (VTs), disease caused by non-vaccine types (NVTs) has steadily increased partially offsetting the total reduction in disease [13–15]. At the same time, pneumococcal carriage rates have remained static due to serotype replacement. Two mechanisms have been suggested for serotype replacement: lineage replacement (NVT lineages expand to fill the vacant ecological niche caused by the removal of VT lineages from the population) and within-lineage NVT expansion (NVT members of a lineage increase in prevalence as VT members decline) [16, 17]. Both mechanisms, in particular lineage replacement, may potentially lead to an increase in previously rare or unobserved serotypes as well as the evolution of novel hybrid serotypes through recombination with other pneumococci or other streptococcal species [8, 18, 19]. Therefore, it is important to understand and monitor the true level of pneumococcal capsular diversity, especially as isolates and genomes from previously understudied regions become increasingly available through projects such as the Global Pneumococcal Sequencing (GPS) project (https://www.pneumogen.net/gps/).

The large number and global distribution of the genomes in the GPS dataset provides an unprecedented opportunity to attempt to identify novel *cps* loci using a dataset consisting of 18 398 genomes which includes 66 of the ~100 described serotypes. The aim of this study was to screen for putatively novel *cps* loci and further characterize any putative novel *cps* loci identified so as to produce a list of candidates for further analysis.

## Methods

### Isolate collection

**Fig. 1.**
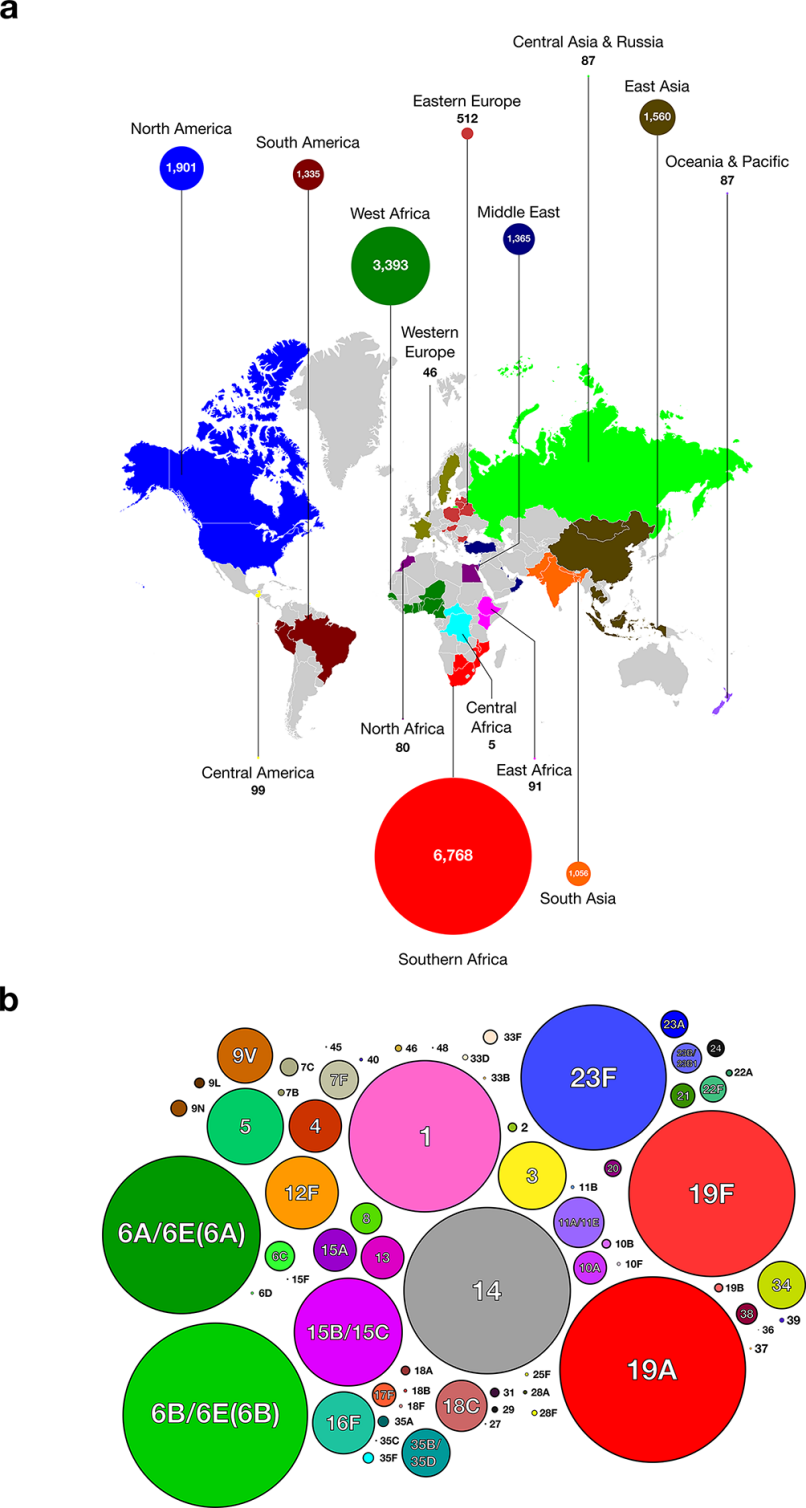
(a) World map showing the global distribution of the samples in the study dataset. (b) Bubble plot showing the 66 serotypes included in the dataset (the area of each circle is proportional to the number of samples).

Isolates in the GPS collection were collected, cultured and sequenced as previously described [20]. A total of 18 768 sequenced genomes were available for inclusion in this study (August 2017). Each genome was assigned to a supraregion based on the country of isolation (Fig. 1a and Supplementary File 2). Serotypes were initially assigned to the isolates on the basis of standard experimental methods including Quellung, latex agglutination and PCR-based arrays.

### Serotypes and lineages

*In silico* serotypes were assigned to the genomes using SeroBA version 1.0.0 [21], a recently described method for determining serotypes based on whole genome sequencing (WGS). Only serotypes represented by >5 genomes were included, resulting in the exclusion of 14 uncommon serotypes: 7A, 9A, 10C, 10X, 11C, 17A, 25A, 32F, 33A, 33C, 39X, 41F, 42 and 43 (*n*=44). In addition, 178 non-typeable or non-encapsulated (NT) [22] and 148 un-typeable (a serotype prediction was unable to be made) genomes were also removed from the dataset. Genomes were predicted as un-typeable due to insufficient read coverage over the reference sequence (<10×; *n*=48; read coverage across the entire genome was low for these samples), the presence of the *nspA*/*pspK* NT variant [18, 23] not included in SeroBA’s list of references (*n*=99) and failures due to software errors (*n*=1). Thus, only 49/18 768 (0.003 %) genomes in the initial dataset were discarded because no serotype could be inferred either due to low mapping coverage (*n*=48) or software failures (*n*=1). This left 18 398 genomes covering 66 serotypes and *cps* variants (Table S1). The variant *cps* loci 23B1 and 6E were included with their related serotypes (23B and 6A or 6B depending on the nucleotide sequence of the *cps* locus background) in the analyses. Due to the uncertainty over the ability of SeroBA to differentiate these serotypes from other highly similar serotypes, the recently described serotypes 11E and 35D were included in the serotype 11A and 35B datasets, respectively.

Recently, 621 pneumococcal lineages (GPSC1–GPSC621) were defined based on a collection of 20 027 genomes from the GPS dataset and other previously published datasets using a Kmer-based clustering tool PopPUNK [24, 25]. These lineages were assigned to the genomes included in this study and used in place of sequence type (ST) or clonal complex (CC).

### Mapping and phylogenetics

For each serotype, sequence reads were mapped back to the appropriate reference (Table S2) using BWA mem version 0.7.17 (minimum and maximum insert sizes of 50 and 1000, respectively) to create *cps* locus alignments. SNPs were called and annotated using SAMtools version 1.2 mpileup [26] and BCFtools version 1.2 as previously described [27] using the following criteria: two reads spanning the base in each direction with at least 75 % consensus, a minimum base call quality of 50 and a minimum root squared mapping quality of 30. Alignments containing SNP sites were extracted from the resulting alignments using SNP-sites version 2.4.1 [28] and maximum-likelihood phylogenies were constructed using FastTreeMP version 2.1.9 [29] (nucleotide general time-reversible model) and visualized and annotated with metadata using iCANDY (https://github.com/simonrharris/iCANDY) and Inkscape (http://www.inkscape.org/).

### Sequence analysis

Average nucleotide identity (ANI) values were calculated for longer sequences (i.e. complete *cps* loci) using FastANI version 1.0 [30] with a fragment length of 1000 bp and a minimum number of fragments equal to 50. Nucleotide identity (NI) values were calculated using cd-hit version 4.6 [31] using the alignment criteria of 90 % identity and 90 % alignment. blast [32] was used to generate comparisons between reference and putative novel *cps* locus nucleotide sequences, and comparison figures were generated using the Artemis Comparison Tool (ACT) [33] and edited in Inkscape.

### Identification of novel *cps* loci

**Fig. 2.**
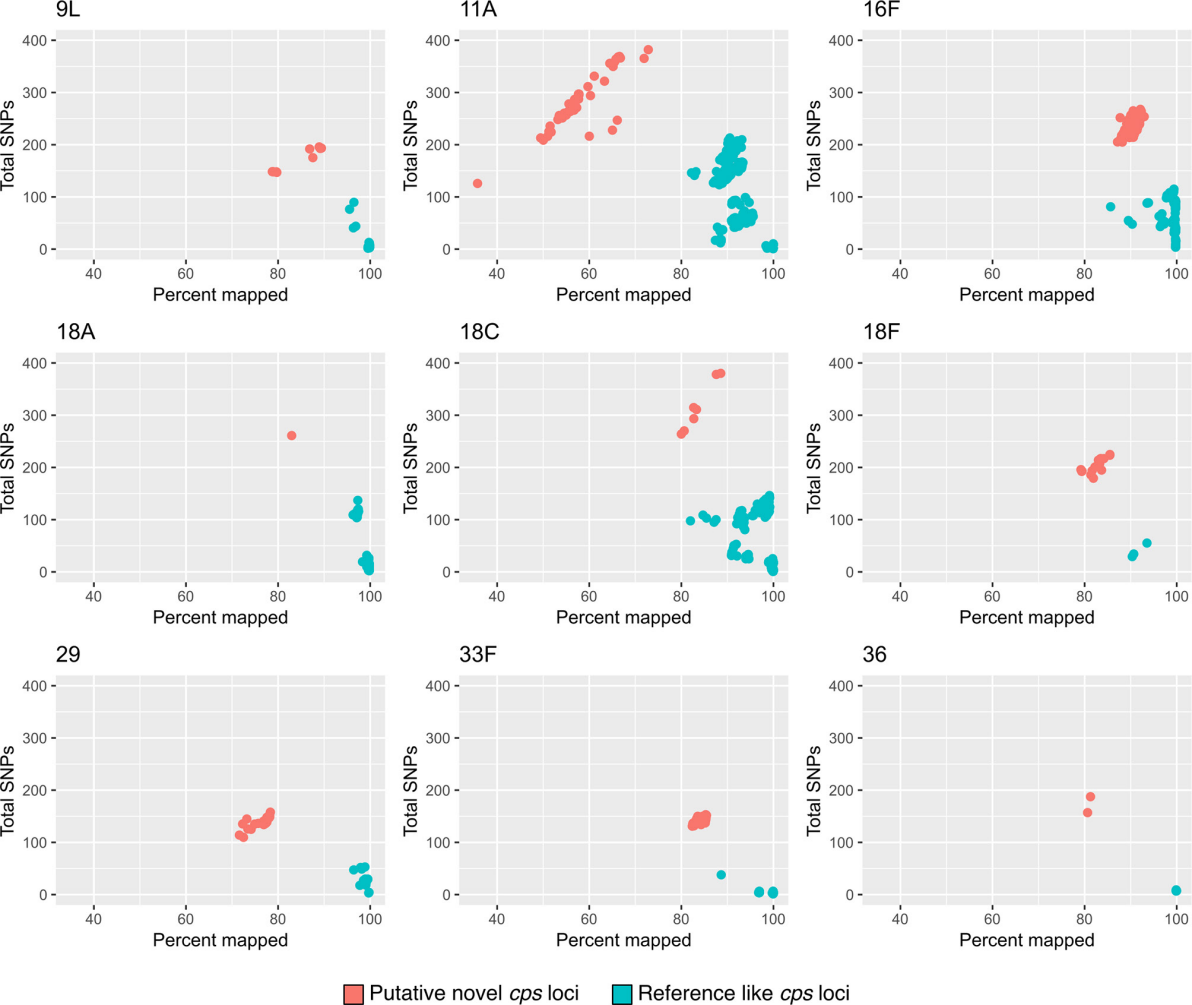
Identification of putative novel *cps* loci; plots of total SNPs against the percentage of the serotype reference covered by sequence reads are shown for nine serotypes with putative novel *cps* loci (highlighted in red).

The distribution of SNPs and the percentage of the reference mapped by sequence reads were assessed for each serotype; genomes with *cps* loci that had a higher number of SNPs compared to other genomes predicted to have that serotype, as well as a lower percentage of the reference covered by sequence reads, were considered to be putative novel *cps* loci and investigated further (Fig. 2).

## Results

In total, 18 398 genomes from 54 countries and 15 supraregions were included in this study (Fig. 1a). The supraregions most represented in the dataset were Southern Africa (*n*=6768), West Africa (*n*=3393), North America (*n*=1901), East Asia (*n*=1560) and the Middle East (*n*=1365); these regions included the key pre- and post-vaccine collections from South Africa, The Gambia, USA, Malawi and Israel. Approximately 60 % of the isolates were from invasive disease whilst the rest were from carriage or unknown sources.

A total of 66 serotypes and *cps* variants were included in this study (Fig. 1b). Of the genomes included in the analyses, 44.2 % (*n*=8136) had serotypes targeted by the 10-valent Pneumococcal Conjugate Vaccine (PCV10) and 60.8 % (*n*=11 188) serotypes targeted by the 13-valent PCV13. The serotypes not targeted by either of the currently available PCVs with the most representation were serotypes 15B/15C (*n*=801), 12F (*n*=546), 16F (*n*=463) and 11A/11E (*n*=375). The screen of 18 398 genomes revealed that 476 (2.6 %) had putative novel *cps* loci, of which there were nine examples (Table 1, Fig. 2 and Table S3).

**Table 1.** Summary of putative novel *cps* loci identified in this study Putative novel *cps* loci (in alphanumerical order by related serotype).

Putative *cps* locus	Genomes (*n*)	GPSC lineages (*n*)	Supraregions (*n*)	Countries (*n*)	Carriage (*n*)	Disease (*n*)	Collection dates
9X	9	6	3	5	5	4	2007–2014
11X	44	5	3	4	43	1	2000–2017
16X	338	11	2	6	251	72	2003–2016
18X1	1	1	1	1	1	0	2014
18X2	7	4	3	5	2	5	2006–2016
18X3	16	4	5	7	4	12	1999–2013
29X	29	5	2	5	7	20	2000–2015
33X	30	3	7	10	10	20	2003–2015
36X	2	2	2	2	2	0	2009

### 9X

**Fig. 3.**
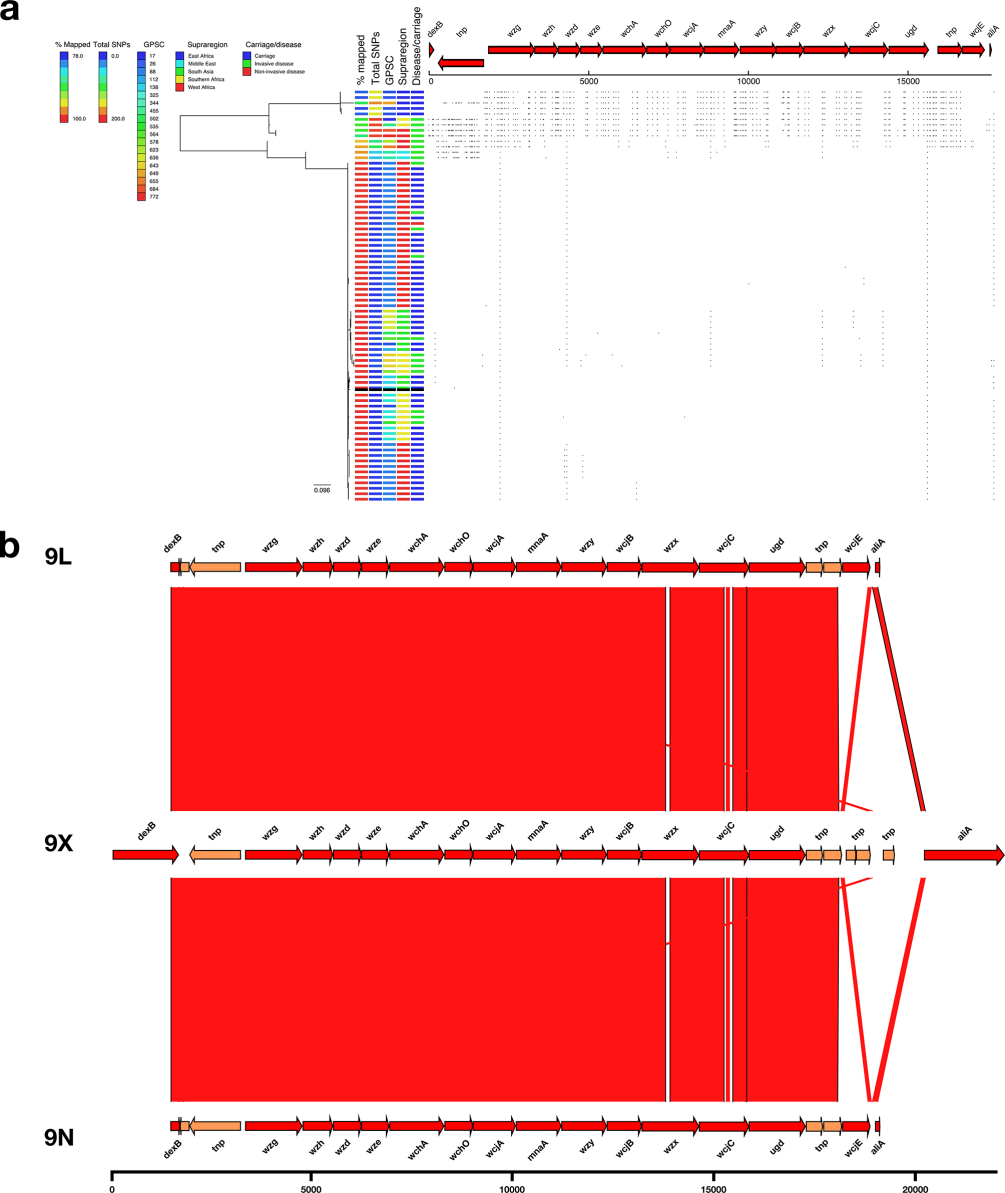
(a) Phylogeny and SNP map of serotype 9L. (b) Comparison of the putative serotype 9X sequence and the serotype 9L and 9N reference sequences.

Analyses of 75 serotype 9L genomes revealed nine with a distinct SNP profile (mean 171.1 SNPs versus mean 8.51 SNPs; Fig. 2) hereafter referred to as serotype 9X (Fig. 3a). The nine isolates were collected in Africa (Ethiopia, Mozambique, South Africa, Ghana and Nigeria), belonged to six different lineages (GPSC17, GPSC26, GPSC455, GPSC649, GPSC684 and GPSC772) and were observed in both invasive disease and carriage. A closer examination of the blast comparison of the serotype 9X *cps* locus compared to serotypes 9L and 9N revealed that whilst the majority of the *cps* locus closely resembled that of serotype 9L (ANI=97.4 %), divergent alleles (<96 % NI) were observed in the *wzh* (90.85 % NI), *wze* (95.91 % NI), *wzy* (94.65 % NI) and *wcjB* (95.86 % NI) genes. Additionally, *wcjE*, which is frameshifted in both serotypes 9L and 9N, was replaced by a transposon in the serotype 9X *cps* locus (Fig. 3b).

### 11X

**Fig. 4.**
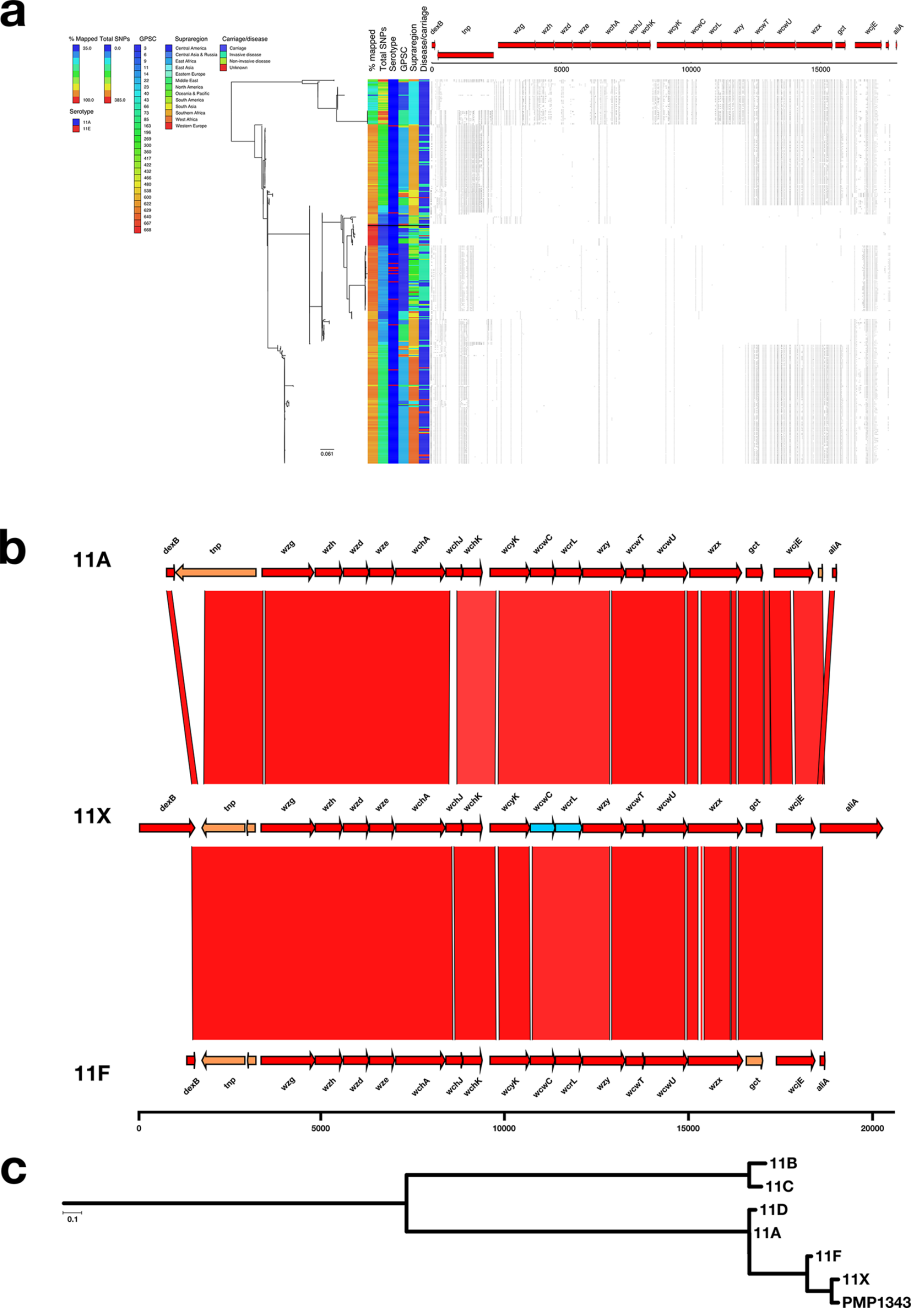
(a) Phylogeny and SNP map of serotype 11A. (b) Comparison of the putative serotype 11X sequence and the serotype 11A and 11F reference sequences. (c) Phylogeny containing the serogroup 11 references, serotype 11X and PMP1343.

Examination of the serotype 11A/11E genomes revealed that 44/375 had a divergent *cps* locus (11X) with much lower reference mapping scores (mean 58.09 % versus mean 92.06 %; Fig. 2) and a higher number of SNP sites (mean 281.39 versus mean 116.79) when compared to the rest of the serotype 11A/11E genomes (Fig. 4a). Comparison of the 11X nucleotide sequence with the serotype 11A reference (ANI=93.9 %) showed that 11X contains divergent alleles for *wcwC* (acetyltransferase; 90.31 % NI) and *wcrL* (glycosyltransferase; 85.48 % NI).

### 16X

**Fig. 5.**
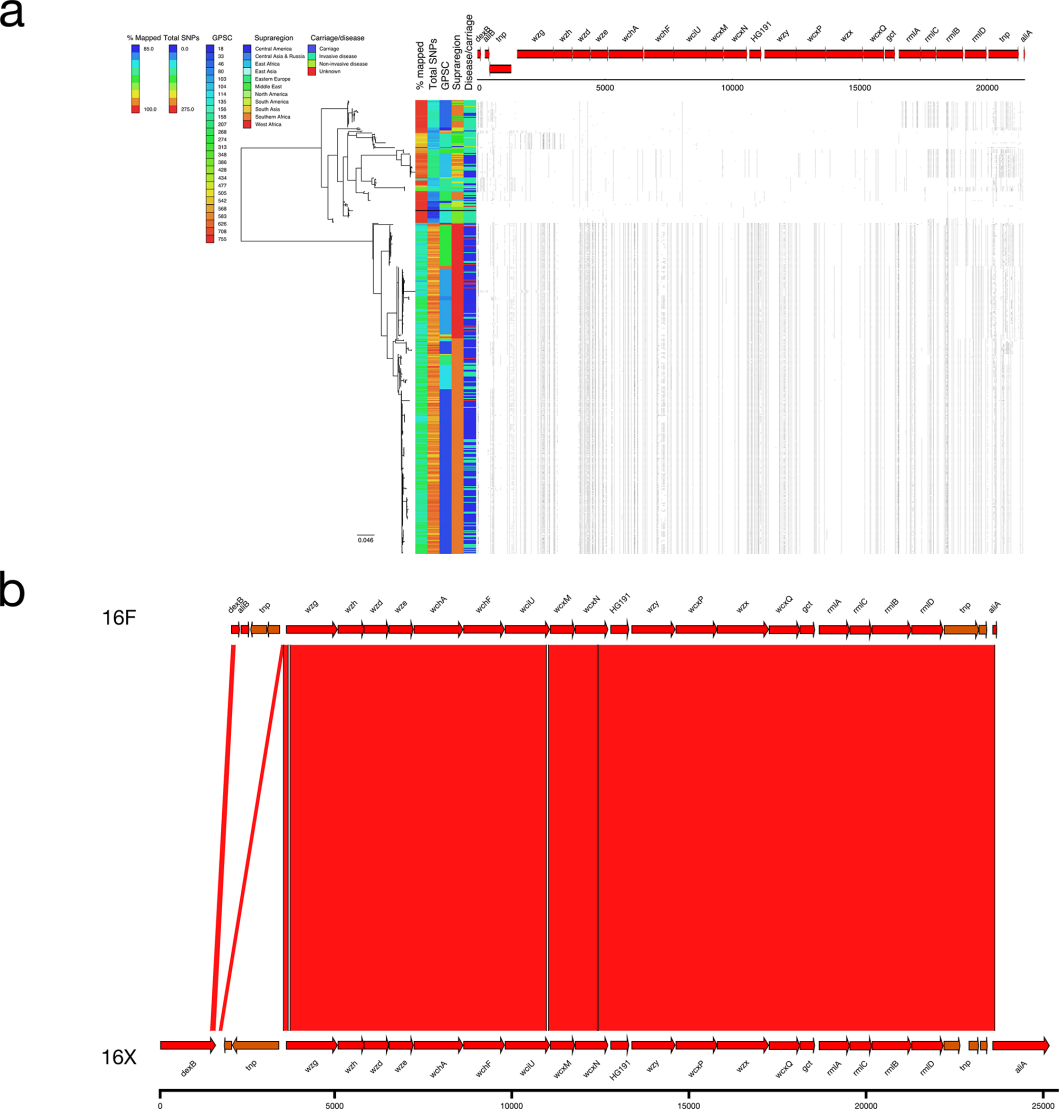
(a) Phylogeny and SNP map of serotype 16F. (b) Comparison of the putative serotype 16X sequence and the serotype 16F reference sequence.

Two distinct clades were observed in the serotype 16F phylogeny with clear separation between reference-like sequences (mean 64.1 SNPs; *n*=125) and a clade with a divergent serotype 16X *cps* locus (mean 240.9 SNPs; *n*=338; ANI compared to 16F: 97.4 %; Fig. 5a). All of the serogroup 16X pneumococci were collected in Southern and West Africa (The Gambia, Malawi, South Africa, Ghana and Cameroon) and were from 11 GPSC lineages. The majority (74.3 %) of the isolates were from carriage while the remaining isolates were from invasive disease or unknown sources. Divergent alleles were present in the *wzh* (82.79 % NI), *wzd* (88.17 % NI), *wze* (94.15 % NI) and *wchF* (95.40 % NI) genes.

### 18X1 and 18X2

**Fig. 6.**
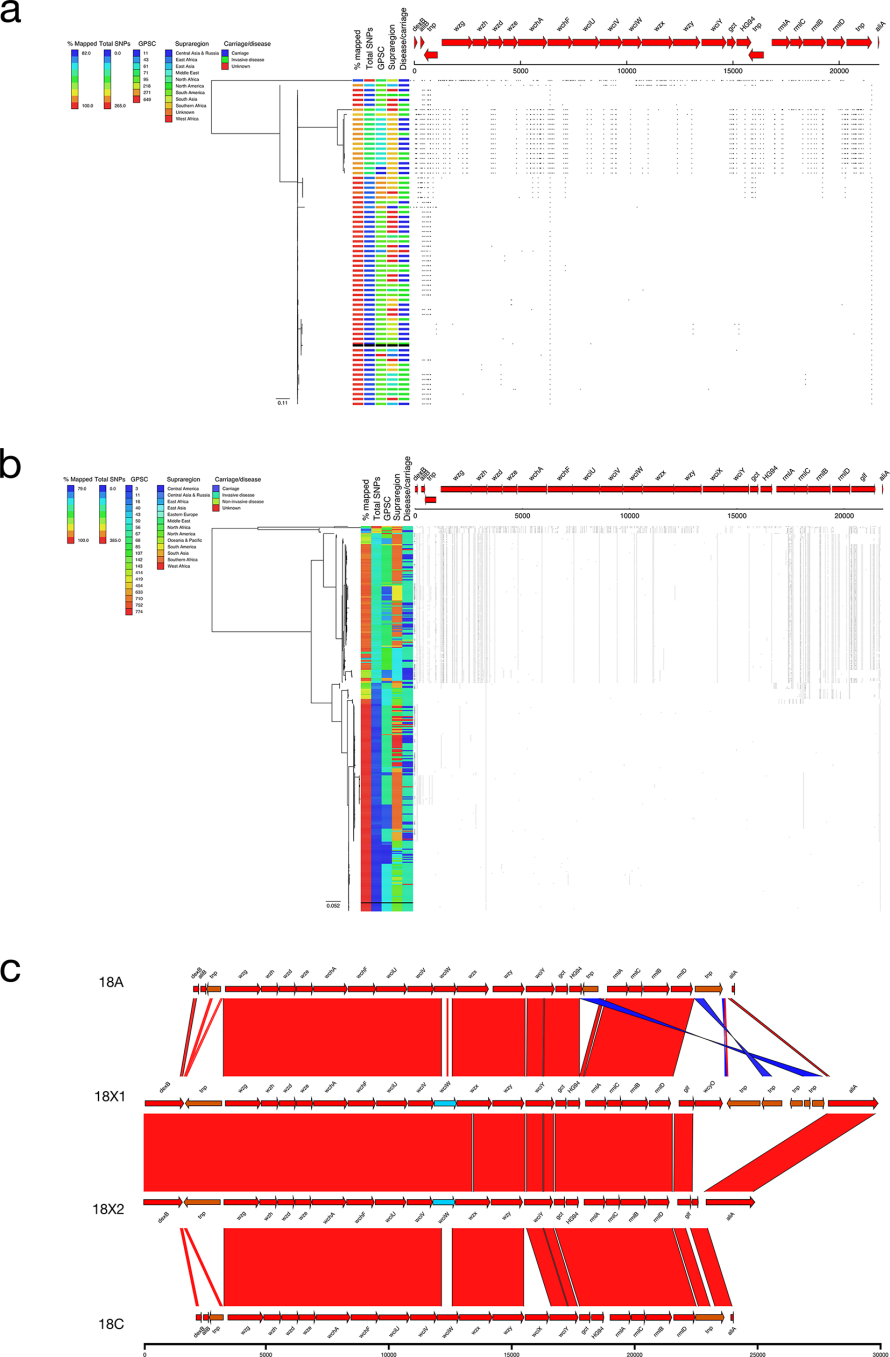
(a) Phylogeny and SNP map of serotype 18A. (b) Phylogeny and SNP map of serotype 18C. (c) Comparison of the putative serotype 18X sequences and the serotype 18A and 18C reference sequences.

A single genome typed as serotype 18A had a putative novel *cps* locus (ANI=95.85 %; 261 SNPs versus mean 33.9 SNPs; Fig. 2). This putative sequence, 18X1, was collected in India in 2014 from a nasopharyngeal swab and was from GPSC71. Seven serotype 18C genomes also had a putative novel *cps* locus, 18X2 (ANI=96.83 %; mean 315.9 SNPs versus mean 55.0 SNPs; Fig. 2). Most (5/7; 71.4 %) of the serotype 18X2 genomes were collected in South Asia (India, Pakistan and Bangladesh) and belonged to four GPSC lineages (GPSC85, GPSC142, GPSC454 and GPSC752). A comparison of the 18X1 and 18X2 sequences revealed that they share a high level of similarity (ANI=99.10 %; Fig. 6) although 18X1 appears to have recombined with serotype 21 at the 3′ end (Fig. S1). Examination of the 18X1 and 18X2 *cps* locus revealed that there were divergent alleles for *wchF* (94.54 % NI), *wciU* (95.60 % NI), *wciW* (81.90 % NI) and *rmlA* (91.03 % NI). Additionally, *glf* (frameshifted in 18X2) was present in 18X1 and 18X2 but not present in either serotypes 18A or 18C.

### 18X3

**Fig. 7.**
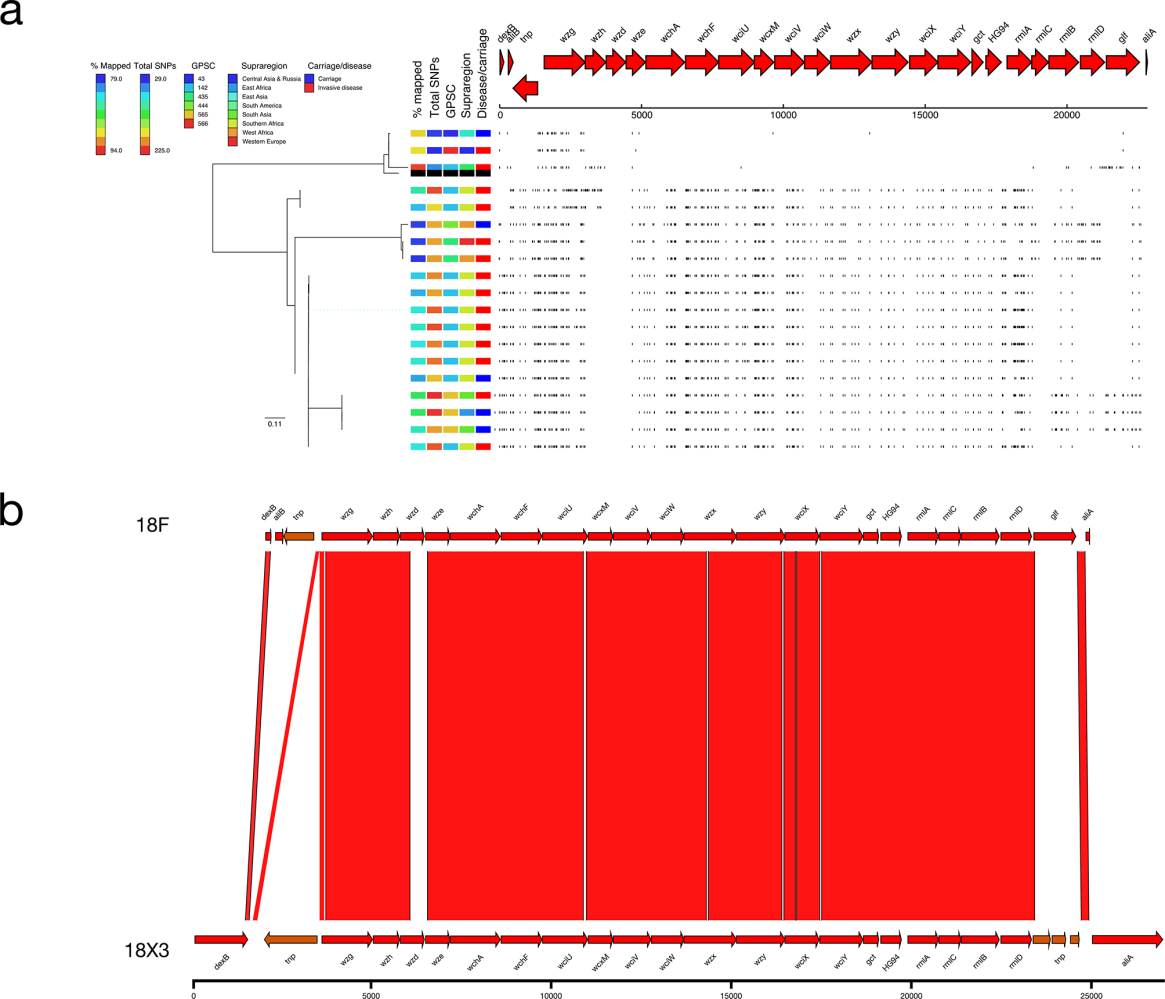
(a) Phylogeny and SNP map of serotype 18F. (b) Comparison of the putative serotype 18Xii sequence and the serotype 18F reference sequence.

The majority (16/19; 84.2 %) of the genomes typed as serotype 18F had a putative novel *cps* locus (ANI 96.13 %) with a distinct SNP profile (mean 203.34 SNPs versus mean 39.33 SNPs; Figs 2 and 7a). These serotype 18X3 genomes were primarily collected in Africa (Ethiopia, Malawi, South Africa and The Gambia; *n*=13) although single isolates were collected in Bangladesh, France and Pakistan. The majority (12/16; 75.0 %) were from invasive disease and a single lineage, GPSC142 (10/16; 62.5 %). Divergent alleles for *wzh* (93.17 % NI), *wzd* (76.91 % NI), *wze* (83.92 % NI), *wchF* (94.71 % NI), *wcxM* (94.76 % NI) and *rmlA* (92.99 % NI) were present in the 18X3 *cps* locus, and *glf* was replaced with a transposon (Fig. 6b). Comparison of the divergent *wzh*, *wzd* and *wze* genes to other serotype sequences revealed that there was a high level of similarity (97.40 % NI) between these alleles and those of serotype 45.

### 29X

**Fig. 8.**
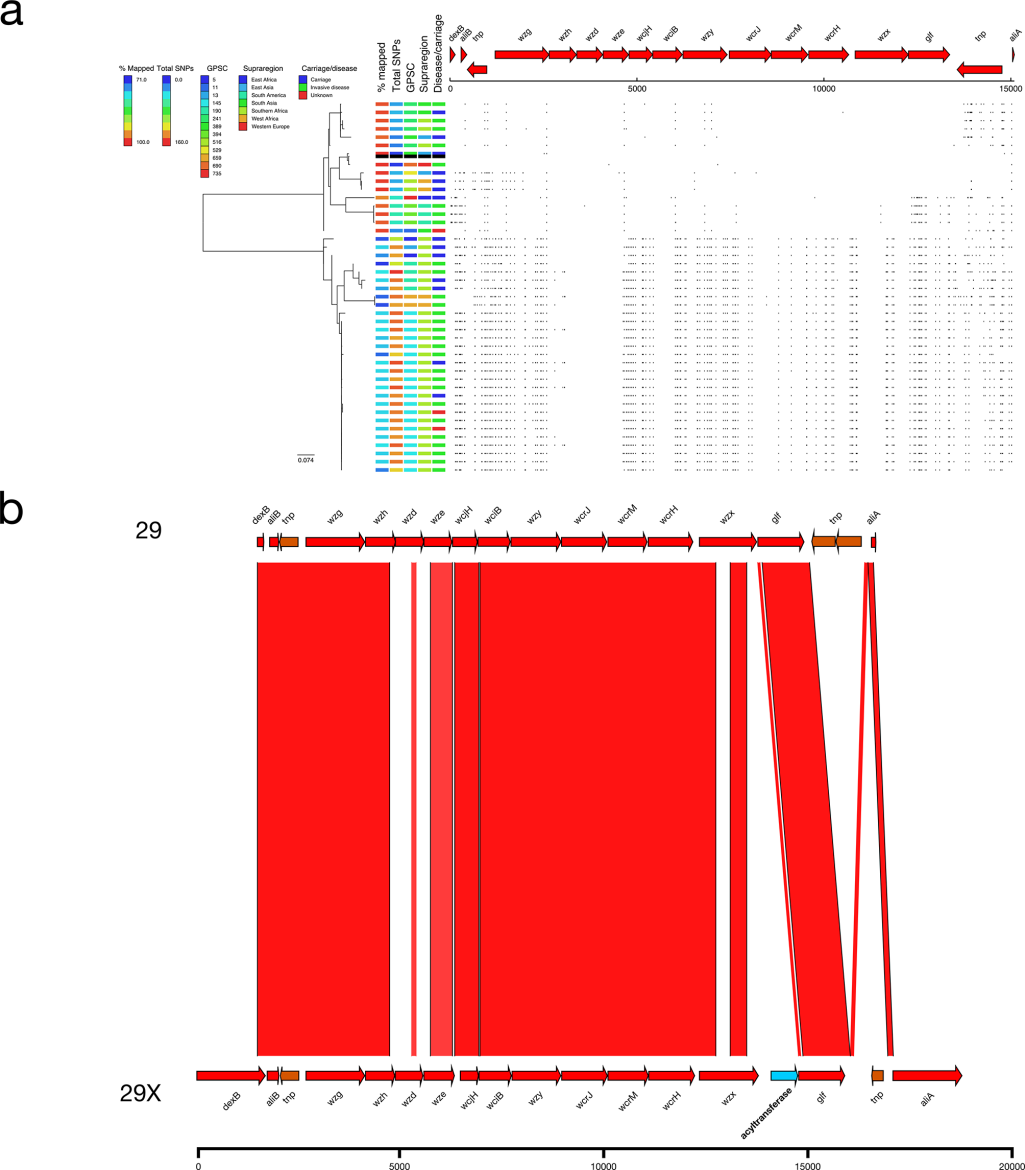
(a) Phylogeny and SNP map of serotype 29. (b) Comparison of the putative serotype 29X sequence and the serotype 29 reference sequence.

In total, 29/45 genomes typed by SeroBA as serotype 29 had a distinct SNP profile (mean 139.51 SNPs; Fig. 2) compared to the reference-like serotype 29 *cps* loci (mean 28.63 SNPs). All of these serotype 29X pneumococci were collected in Africa (Malawi, Mozambique. South Africa, Benin and Ghana), primarily from invasive disease (20/29; 69.0 %) and comprised several GPSC lineages (Fig. 8a). The ANI of the 29X *cps* locus was 95.8 % compared to the serotype 29 reference with highly divergent alleles observed for *wzh* (83.61 % NI), *wzd* (<70 % NI), *wze* (77.92 % NI) and *wzx* (79.66 % NI). Comparison of the *wzh*, *wzd* and *wze* nucleotide sequences to other serotypes identified the equivalent genes in serotype 12F as being highly similar (98.14 % NI). Additionally, the serotype 29X *cps* locus has an extra frameshifted *wciG* (acetyltransferase) located between *wzx* and *glf* (Fig. 8b), which is very similar (99.40 % NI) to *wciG* from serotypes 32F and 35F where the gene is functional.

### 33X

**Fig. 9.**
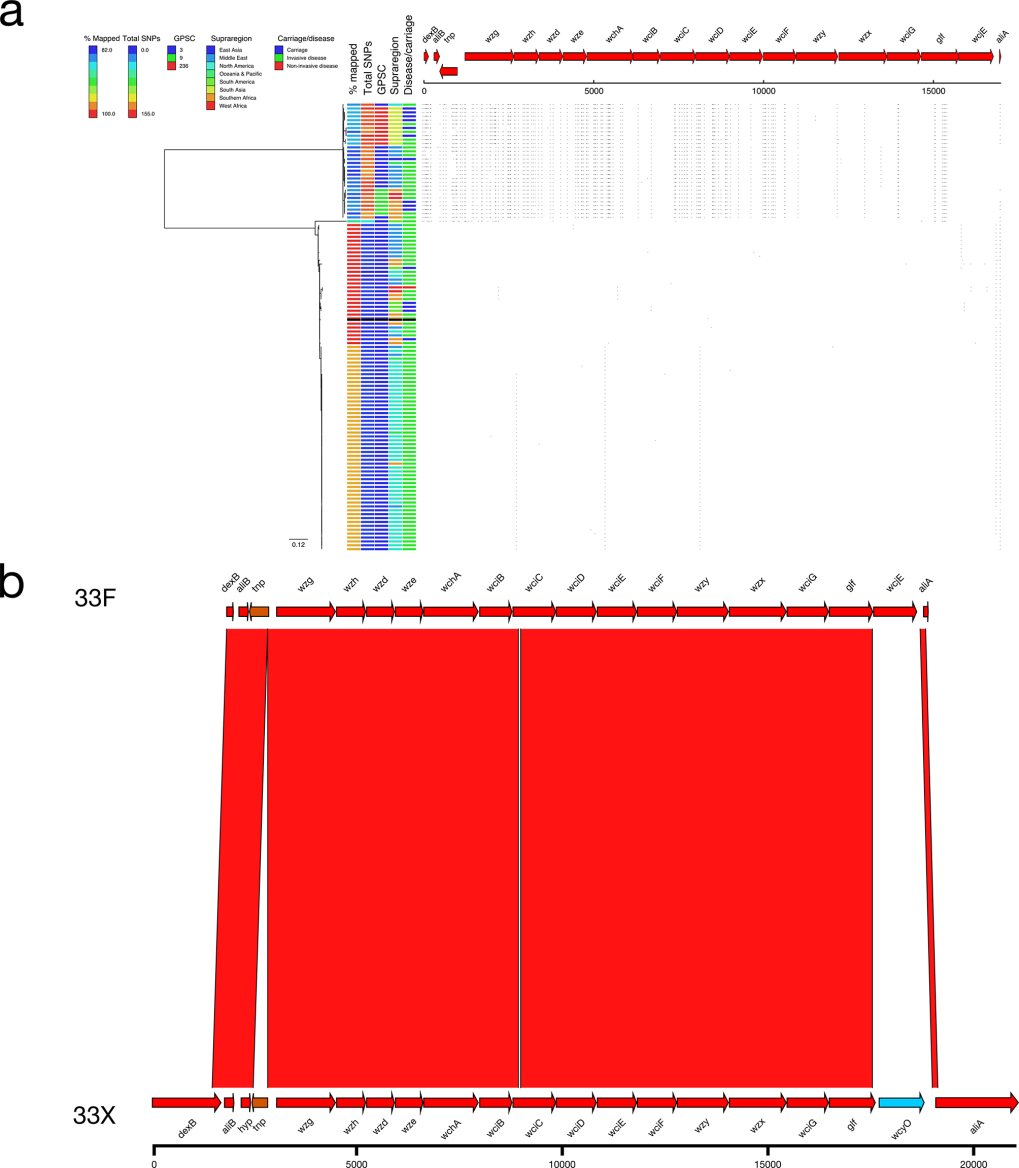
(a) Phylogeny and SNP map of serotype 33F. (b) Comparison of the putative serotype 33X sequence and the serotype 33F reference sequence.

In total, 115 serotype 33F pneumococci were identified in the dataset and, of these, 30 had a distinct SNP profile (mean 143.27 SNPs versus mean 4.84 SNPs; ANI=96.50 %; Figs 2 and 9a). These serotype 33X isolates were collected in ten different countries in seven different supraregions, suggesting a global distribution for this putative novel *cps* locus. The majority were collected from cases of invasive disease (20/30; 66.7 %) and the genetic lineage of the isolates was conserved with 23/30 (76.7 %) coming from GPSC3 or GPSC236 (Fig. 9b). The 33X *cps* locus was primarily 33F-like but divergent alleles were present for *wciG* (89.46 % NI) and *glf* (89.46 % NI) and the frameshifted *wcjE*, which differentiates serotypes 33A and 33F, was replaced by a frameshifted *wcyO,* which was highly similar to the non-frameshifted 33C *wcyO* (99.27 % NI).

### 36X

**Fig. 10.**
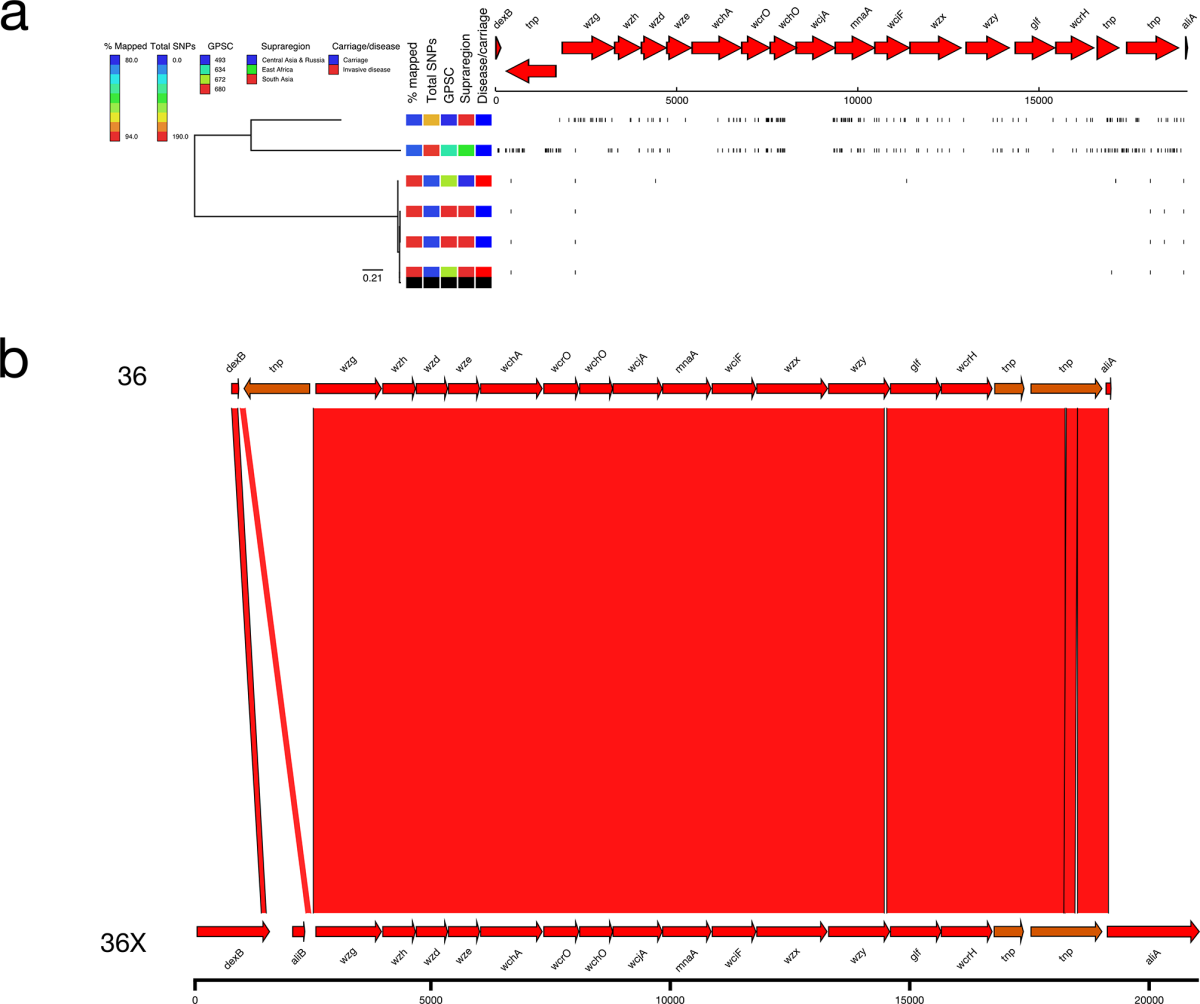
(a) Phylogeny and SNP map of serotype 36. (b) Comparison of the putative serotype 36X sequence and the serotype 36 reference sequence.

A small number of genomes in the GPS dataset (*n*=6) were typed as serotype 36, and of these, two had a divergent SNP profile (mean 171.50 SNPs versus mean 6.75 SNPs; ANI=96.77 %; Figs 2 and 10a). The serotype 36X pneumococci were collected in Ethiopia and Nepal and were both from carriage (Fig. 10a). The serotype 36X *cps* locus has divergent alleles for the glycosyltransferases *wchO* (92.12 % NI) and *wcjA* (85.36 % NI) when compared to the reference (Fig. 10b). A GenBank query revealed that the closest matches for *wchO* (98.06 % NI) and *wcjA* (95.67 % NI) were from other streptococcal species such as *S. mitis* and *S. pseudopneumoniae *, suggesting that the serotype 36X *cps* locus has recently undergone a recombination event.

## Discussion

The aim of this study was to screen the GPS dataset and identify putative novel *cps* loci that may encode novel serotypes. The very large number of genomes and broad geographical coverage of the dataset enabled the first detailed sequence-based analysis of the majority of previously identified serotypes. Other studies have analysed large numbers of pneumococcal genomes (*n* =~5000) but, apart from the more common serogroups such as 6 and 19, the number of genomes analysed for many of the serotypes was relatively small [8, 19]. This study revealed that despite the large number of genomes (and serotypes) included, the number of putative novel *cps* loci was very small: of the 18 398 genomes included, only 476 (2.6 %) had putative novel *cps* loci comprising nine different examples. This was perhaps unexpected given the number and diversity of genomes included; to date nearly 100 serotypes have been identified, with several discovered in the past decade, suggesting that the total number of pneumococcal serotypes was much higher. In fact, the results of this study suggest that we are now close to discovering the total amount of extant global pneumococcal serotype diversity. However, 30 serotypes were not included in this study due to them either not being present in the GPS dataset (*n*=16) or else present in fewer than five genomes (*n*=14). The pubMLST *S. pneumoniae* isolates database (https://pubmlst.org/spneumoniae/) which contains >40 000 records, was queried for the prevalence of the excluded serotypes: only serotype 24F (*n*=214) was found in more than 50 records, suggesting that the missing serotypes could be considered uncommon. However, these may also be a source of novel *cps* loci so future work should include collecting as many examples of each rare serotype to determine the diversity of their *cps* loci.

Despite the comparatively small number of genomes with putative novel *cps* loci, there was a surprising amount of diversity amongst the genomes with these *cps* loci, especially with respect to geography, genomic lineage and their prevalence in both carriage and disease. Pneumococci with putative novel *cps* loci were found in nine of the 15 supraregions assigned in this study, with nearly every continent represented. Seven of the nine putative novel *cps* loci were present in both Southern and West Africa, suggesting that future studies including African isolates may reveal additional novel *cps* loci. The most represented putative novel *cps* locus in the dataset was 16X (338/446 genomes), probably due to the high prevalence of serotype 16F (the majority of which had the 16X cps locus) in South Africa and Gambia, which both contributed large numbers of isolates to the GPS project. The most globally distributed novel *cps* locus was 33X as it was found in seven supraregions comprising ten different countries. There were 40 different GPSC lineages, comprising a large amount of the diversity in the genomic background of the pneumococci, with putative novel *cps* loci and, on average, each putative novel *cps* locus was found in four to five GPSC lineages. With the exception of serotypes 18X1 and 36X, which were only present in one and two genomes respectively, all of the putative novel *cps* loci were found in both carriage and IPD. Serotypes 18X2 (5/7; 71.4 %), 18X3 (12/16; 75 %), 29X (20/29; 70 %) and 33X (20/30; 67 %) were more commonly found in IPD isolates whilst serotype 11X was almost exclusively found in carriage (43/44; 97.7 %). Gladstone *et al*. (Gladstone accepted by EbioMed) calculated invasiveness using odds ratios for some of the serotypes associated with putative novel *cps* loci: serotype 11A was significantly associated with carriage whilst serotypes 18C and 33F were significantly associated with disease. The carriage/disease distributions of the putative novel *cps* loci 18X2 and 33X matched those of the related serotypes whilst 11X, being more commonly found in disease, did not match the distribution of serotype 11A.

The introduction of PCVs has had a dramatic effect on the global pneumococcal population with VTs decreasing in prevalence in both invasive disease and carriage. At the same time disease caused by NVTs has increased along with the related stable carriage rates. Due to this ecological perturbation, it is possible that previously rare or unknown serotypes may increase in circulation allowing them to be more likely to be detected. The evidence in this study for this is relatively limited given the small numbers and wide geographical distribution of most of the genomes with putative novel *cps* loci. However, 182/338 genomes with the 16X *cps* locus from the same lineage, GPSC33, and collected in South Africa increased from 42 isolates pre-vaccine to 99 post-PCV, although this increase in prevalence may be due to a clonal expansion rather than because of the novel *cps* locus. All of the above observations highlight the necessity for continued global WGS surveillance of important pathogens such as pneumococcus.

A number of methods were used to serotype the isolates collected as part of the GPS project, with Quellung or a combination of Quellung and PCR or Quellung and microarray, accounting for the majority (10 728/12 369; 86.7 %) of the phenotypic serotypes provided at the time of sequencing. A significant number of the isolates included in this study lacked phenotypic data for the serotype (6013/18 382; 32.7 %).

All of the serotype assignment for the genomes included in this study was performed by WGS using SeroBA and showed good concordance with the phenotypic methods such as Quellung or Latex agglutination (Table S1). Using the results of the WGS-based serotyping also allowed genomes lacking phenotypic classification, which otherwise would have been excluded, to be included in the analyses. This highlighted that it is now much simpler and faster to serotype pneumococci using the genome sequence and only perform further phenotypic classification in the event of one of the WGS-based tools failing due to a misassembly or else a *cps* locus distinct from the set of reference genomes.

Various different mechanisms are responsible for differences between the polysaccharides produced by the pneumococcal *cps* locus and many of these were identified in this study. Previous studies have shown that small changes to *cps* locus genes, such as single amino acid changes as in serotypes 6A and 6B, may lead to differences in the polysaccharide structure whilst sequence divergence of 5 %, as in the case of the *cps* locus variant 6E (6Bii) and serotype 6B, or the 30 % divergence in homology between serotype 23B and the 23B1 variant, may not lead to a change in the structure of the polysaccharide capsule [34, 35]. In a number of serotypes, such as 9A, 22A and 33F, the differences between the polysaccharide capsules produced by highly similar *cps* loci (e.g. 9V, 22F and 33A) are due to frameshifts in genes at the 3′ end of the cps locus such as *gct* and *wcjE*. This is due to mutations in these genes not affecting the genes at the 5′ end of the *cps* locus, which allows disruptive mutations which would be lethal if they occurred elsewhere in the operon [10, 36]. Different polysaccharide structures may also occur when these genes are replaced by transposons such as in serotype 16F. Examples of both of these mechanisms were observed in this study. Serotype 11X, like 11A, has an intact *gct* which correlates with the presence of gro-IP in the polysaccharide (*gct* is frameshifted and thus inactive in serotype 11F) [12]. The presence of the functional *glf* in 18X1 suggests that galactofuranase may be present in the polysaccharide and absent in 18X2. The serotype 33C-like frameshifted *wcyO* in serotype 33X has been shown to mediate the same capsular modification as the serotype 33F *wcjE* [37, 38]. Two examples of *wcjE* or *glf* being replaced by transposons were observed in serotypes 9X and 18X3, respectively. The replacement of *glf* by a transposon in the 18X3 *cps* locus suggests that galactofuranase may be absent in the 18X3 polysaccharide capsule.

Amino acid changes to acetyltransferases and glycosyl transferases as well as additional copies of these genes may change the acetylation profile, repeat patterns and sugar constitution of the polysaccharide capsule. Examples of changes to acetyltransferases were found in many of the putative novel *cps* loci. For instance, amino acid changes in *wcwC*, similar to those found in serotype 11X, have been shown to lead to differences in acetylation whilst *wcrL* encodes a glycosyltransferase responsible for different sugars in serotypes 11F (GlcpNAc) and 11A (Glcp) [39]. The complete structure of the serotype 16F polysaccharide is currently unknown but amino acid changes in the glycosyltransferase *wchF* of serotype 16X may change the constituents of the polysaccharide repeat units [11]. Due to recombination between the 18X2 and serotype 21 *cps* loci, the 18X1 *cps* locus also possesses an additional acetyltransferase, *wcyO*, which may lead to differences in acetylation in the polysaccharide whilst the variant acetyltransferase, *wcxM* of serotype 18X3, may also lead to differences in acetylation when compared to serotype 18F. The unique glycosyltransferase *wciW* found in serotypes 18X1 and 18X2, along with amino acid changes in *wchF*, suggests a different pattern of polysaccharide repeat units. The 18X1 and 18X2 *wciU* allele is more similar to the 18C version; this gene is responsible for the addition of Glcp to the polysaccharide capsule in serotypes 18B, 18C and 18F and GlcpNAc in 18A. The serotype 29X *cps* locus has an additional frameshifted *wciG* (acetyltransferase), located between *wzx* and *glf*, which is very similar (99.40 % NI) to *wciG* from serotypes 32F and 35F where the gene is functional. The divergent alleles for the glycosyltransferases *wchO* and *wcjA* in serotype 36X may also lead to changes in the patterns of repeats in the polysaccharide.

Other studies have recently identified new serotypes [7, 8], particularly amongst pneumococci isolated in particular regions such as South East Asia, which is currently underrepresented in this collection. The serotype 11X *cps* locus has been previously identified [19, 40] and has been shown to possess a predominately serotype 11F *cps* locus while phenotypically serotyping, using Quellung and latex agglutination, as serotype 11A. An example of this novel serotype or serotype variant (PMP1343) was made publicly available and was included in a phylogenetic tree along with 11X and the other members of serogroup 11 (Fig. 4c). The close proximity of PMP1343 and 11X confirmed that these are likely to be the same *cp*s locus type as well as showing that the *cps* locus, whilst closely related to serotype 11F, is unique (Fig. 4c). In total, 42/44 serotype 11X pneumococci were collected in Cambodia and Hong Kong, suggesting a primarily Asian or Pacific distribution, in line with the previous findings that pneumococci with this *cps* locus have previously been collected in Fiji, Thailand, Mongolia and Laos [19, 40]. The serotype 33X *cps* locus has recently been found (labelled as serotype variant 33F-1) in pneumococci isolated from healthy children and children with pneumonia in Fiji and Mongolia, respectively [38]. These studies along with the analysis conducted in this paper show that novel serotypes will continue to be discovered as more pneumococci from previously understudied regions are sequenced.

The methodology used in this study was designed to identify highly divergent *cps* loci with different alleles of the same gene as well as additional genes as were identified in serotype 36X. The analyses were not designed to be sensitive to very fine changes in the *cps* loci such as non-synonymous mutations leading to amino acid changes. It is likely that there would be additional diversity in the form of putative novel *cps* loci, when considering small-scale changes such as non-synonymous mutations, that has not been detected in this analysis. One of the aims of this study was to screen a large number of genomes using a simple, robust method that would quickly identify divergent *cps* loci before focusing on genomes with loci of interest. Given the scale of this undertaking and the large number of putative novel *cps* loci identified that will be studied further, it was decided that a finer scale analysis would form part of a subsequent study that will make use of the outputs generated as part of this study. This analysis will be conducted alongside the studies detailing the serological and biochemical characterisation of the putative novel *cps* loci identified in this study.

Examples of pneumococci with each putative novel *cps* locus identified in this study will undergo further analyses including the biochemical elucidation of their polysaccharide structure, confirming that capsules are being produced, i.e. proving that these putative novel *cps* loci are not just defective variants of known *cps* loci, and assessing how these capsules react serologically to known sera using Quellung. Whilst techniques such as GLC and NMR have revolutionized the study of polysaccharide structures, difficulties still remain [10]. In particular, these techniques rely on pure capsular polysaccharides, which depending on the serotype may be difficult to purify due to the inclusion of the covalently linked cell-wall polysaccharide. Some of the putative novel *cps* loci identified are similar to *cps* loci for which the polysaccharide structure is yet to be discerned (serotypes 16F, 29 and 36) so structures for these serotypes will also need to be determined at the same time to establish whether the novel *cps* loci are indeed undescribed.

This study identified nine putative novel *cps* loci in a large, global dataset although the number of genomes with these *cps* loci was comparatively small, suggesting that the majority of pneumococcal serotypes have now been identified. However, given that many of these genomes were from regions such as Africa and South-East Asia where pneumococcal genome sequencing has to date been infrequent, further monitoring and surveillance of pneumococcal *cps* loci coming from these regions is required.

## Data bibliography

1. van Tonder, AJ 2018.

## Supplementary Data

Supplementary File 1

Supplementary File 2

Supplementary File 3
