# Supplementary material for: Putative novel cps loci in a large global collection of pneumococci
Source: Microb Genom. 2019 Jun 11;5(7):e000274. doi: 10.1099/mgen.0.000274 (PMC6700660; doi:10.1099/mgen.0.000274)
Supplement: Supplementary File 1 [file mgen-5-274-s001.pdf]

Supraregion definitions used in this study

North America

Canada, USA

Central America

Guatemala, Trinidad and Tobago

South America

Brazil, Ecuador, Peru

West Africa

Benin, Cameroon, Ghana, Ivory Coast, Niger, Nigeria, Senegal, The Gambia, Togo, West Africa

North Africa

Egypt, Morocco

Central Africa

Central African Republic, DRC

East Africa

Ethiopia, Kenya

Southern Africa

Botswana, Malawi, Mozambique, South Africa

Western Europe

France, Netherlands, Sweden

Eastern Europe

Belarus, Bulgaria, Hungary, Latvia, Lithuania, Poland, Slovenia

Middle East

Israel, Kuwait, Oman, Qatar, Turkey

Central Asia & Russia

Russia

South Asia

Bangladesh, India, Nepal, Pakistan

East Asia

Cambodia, China, Indonesia, Malaysia, Mongolia, Thailand

Oceania & Pacific

New Zealand

Table S2: *cps* locus references

Reference	Other name	Accession number	Year	Country of isolation	PubMed ID
1	519/43	CR931632	1943	Denmark	16532061
2	Pn2L	CR931633	1956	USA	16532061
3	524/62	CR931634	1962	Denmark	16532061
4	600/62	CR931635	1962	Denmark	16532061
5	Ambrose	CR931637	1976	USA	16532061
6A	34351 Rodriques	CR931638	1952	USA	16532061
6B	2616/39	CR931639	1939	Denmark	16532061
6C	CHPA388	EF538714	1999-2002	USA	17576753
6D	MNZ21	HM171374	2008	South Korea	20929956
7B	Johnson	CR931641	1952	USA	16532061
7C	Sutcliff	CR931642	1971	USA	16532061
7F	554/62	CR931643	1962	Denmark	16532061
8	573/62	CR931644	1962	Denmark	16532061
9L	T9233/128/68	CR931646	1968	Denmark	16532061
9N	533/62	CR931647	1962	Denmark	16532061
9V	980/68	CR931648	1968	Denmark	16532061
10A	10061/38	CR931649	1938	Denmark	16532061
10B	423/82	CR931650	1982	Denmark	16532061
10F	34355	CR931652	1956	USA	16532061
11A	1813/39	CR931653	1939	Denmark	16532061
11B	8087/40	CR931654	1940	Denmark	16532061
12A	559/66	CR931658	1966	Denmark	16532061
13	34357	CR931661	1952	USA	16532061
14	34359	CR931662	1952	USA	16532061
15A	389/39	CR931663	1939	Denmark	16532061
15B	7904/39	CR931664	1939	Denmark	16532061
15F	688/63	CR931666	1963	Denmark	16532061
16F	nr.34361	CR931668	1952	USA	16532061
17F	Rose	CR931670	1952	USA	16532061
18A	8609/43	CR931671	1952	Denmark	16532061
18B	1033/41	CR931672	1941	Denmark	16532061
18C	4593/40	CR931673	1940	Denmark	16532061
18F	Gethens	CR931674	1961	USA	16532061
19A	nr. 141/68	CR931675	1968	Denmark	16532061
19B	nr. 4594	CR931676	1971	Germany	16532061
19F	485/61	CR931678	1961	Denmark	16532061
20	34365	CR931679	1937	Denmark	16532061
21	546/62	CR931680	1962	Denmark	16532061
22A	3405/39	CR931681	1939	Denmark	16532061
22F	1772/40	CR931682	1940	Denmark	16532061
23A	1196/45	CR931683	1945	Denmark	16532061
23B	1039/41	CR931684	1941	Denmark	16532061
23F	Dr Melchior	CR931685	1996	Denmark	16532061
24A	2748/40	CR931686	1940	Denmark	16532061
25F	601/62	CR931690	1962	Denmark	16532061
27	nr.34371	CR931691	1956	USA	16532061
28A	1982/45	CR931692	1945	Denmark	16532061
28F	34372	CR931693	1957	USA	16532061

29	34373	CR931693	1952	USA	16532061
31	34374	CR931695	1952	USA	16532061
33B	E294	CR931699	1962	Denmark	16532061
33D	CSF/79	CR931701	1979	India	16532061
33F	3084/37	CR931702	1937	Denmark	16532061
34	676/74	CR931703	1974	Denmark	16532061
35A	1936/39	CR931704	1939	Denmark	16532061
35B	4356/39	CR931705	1939	Denmark	16532061
35C	7765/43	CR931706	1943	Denmark	16532061
35F	361/39	CR931707	1939	Denmark	16532061
36	1095/39	CR931708	1939	Denmark	16532061
37	264/73	CR931709	1973	Denmark	16532061
38	9687/39	CR931710	1936	Denmark	16532061
39	203/40	CR931711	1940	Denmark	16532061
40	Colemore	CR931712	1950	USA	16532061
45	Eddy nr.72	CR931718	1954	USA	16532061
46	Eddy nr.73	CR931719	1954	USA	16532061
48	656/63	CR931722	1963	Denmark	16532061

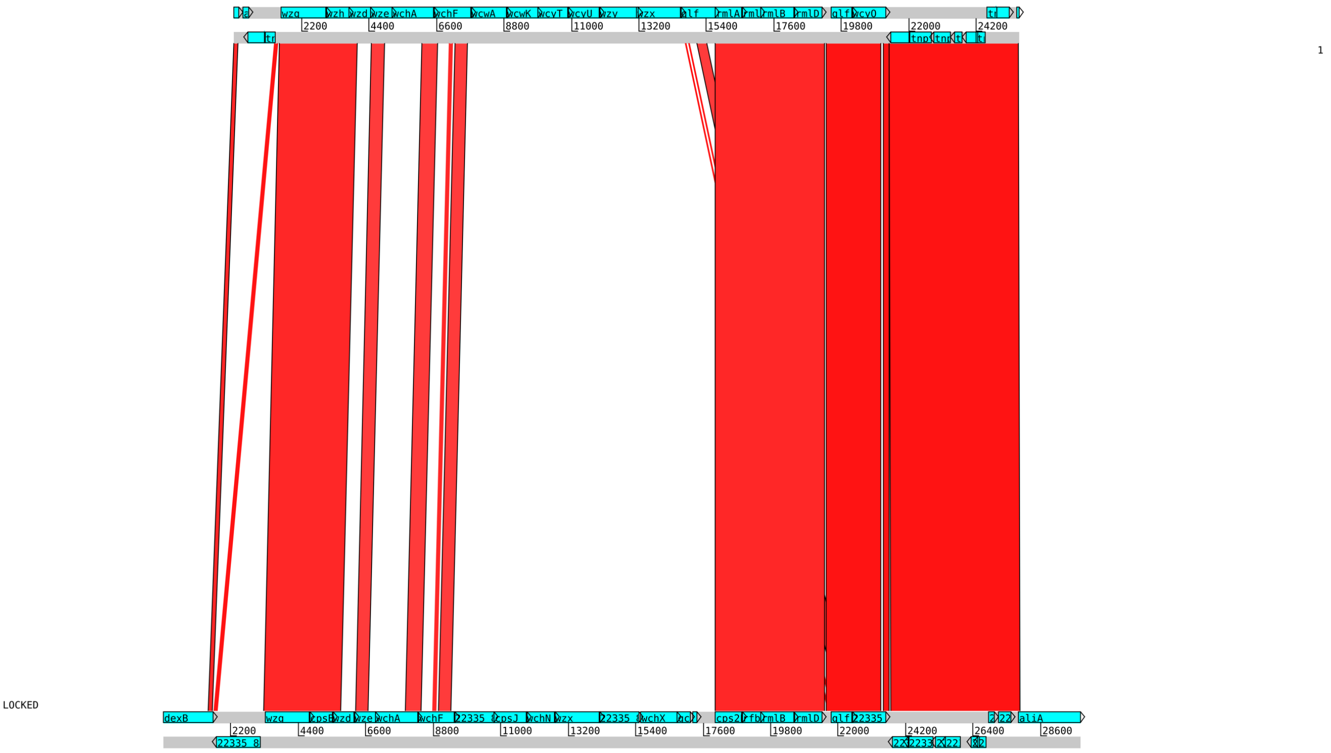

Figure S1: ACT comparison of serotypes 18X and 21; red bars show sequence similarity between the two sequences
