# Supplementary material for: Putative novel cps loci in a large global collection of pneumococci
Source: Microb Genom. 2019 Jun 11;5(7):e000274. doi: 10.1099/mgen.0.000274 (PMC6700660; doi:10.1099/mgen.0.000274)
Supplement: Supplementary File 3 [file mgen-5-274-s003.pdf]

Members of The Global Pneumococcal Sequencing Consortium

Abdullah W Brooks
Alejandra Corso
Alexander Davydov
Andrew Pollard
Anna Skoczynska
Bernard Beall
Betuel Sigauque
Deborah Lehmann
Diego Faccone
Ekaterina Egorova
Elena Voropaeva
Eric Sampane-Donkor
Ewa Sadowy
Godfrey Bigogo
Helio Mucavele
Houria Belabbès
Idrissa Diawara
Jennifer Moïsi
Jennifer Verani
Jeremy Keenan
KL Ravikumar
Leonid Titov
Margaret Ip
Maria-Cristina de Cunto Brandileone
Md Hasanuzzaman
Metka Paragi
Naima Elmdaghri
Nicole Wolter
Noga Givon-Lavi
Özgen Köseoglu Eser
Pak Leung Ho
Patrick E Akpaka
Paul Turner
Paula Gagetti
Peggy-Estelle Tientcheu
Philip E. Carter
Pierra Law
Rama Kandasamy
Rebecca Ford
Sadia Shakoor
Samanta Cristine Grassi Almeida
Samir K. Saha
Sanjay Doiphode
Susan A. Nzenze
Shamala Devi Sekaran (Sekaran SD)
Somporn Srifuengfung
Stephen Obaro
Stuart C Clarke

Tamara Kastrin
Theresa J. Ochoa
Veeraraghavan Balaji
Waleria Hryniewicz
Yulia Urban
